# Arterial cannulation site and ventilation strategy modulate cerebral injury in a rat cardiopulmonary bypass model of Harlequin syndrome

**DOI:** 10.1186/s40635-026-00955-9

**Published:** 2026-09-01

**Authors:** Chadi Aludaat, Gabriel Saiydoun, Saade Saade, Jean-Marc Baste, Vincent Magnan, Fabien Doguet, Fabrice Bauer, Jeremy Bellien, Paul Mulder, Emmanuel Besnier

**Affiliations:** 1 Department of Cardiothoracic Surgery, Rouen University Hospital, Rouen, 76000 France; 2 Normandie Univ, UNIROUEN, INSERM U1096, FHU REMOD-VHF, Rouen, France; 3 Department of Cardiothoracic Surgery, AP-HP, La Pitié-Salpêtrière Hospital, Sorbonne University, Paris, 75013 France; 4 Department of Cardiothoracic Surgery, Strasbourg University Hospital, Strasbourg, 67000 France; 5 Department of Cardiac Surgery, Institut Jacques Cartier, Massy, 91300 France; 6 Université Paris-Saclay, Faculté de Médecine, HPPIT, Pulmonary Hypertension: Pathophysiology and Novel Therapies, Le Kremlin-Bicêtre, 94270 France; 7 Department of Cardiothoracic Anesthesia and Critical Care, Rouen University Hospital, Rouen, 76000 France

**Keywords:** Cardiopulmonary bypass, Harlequin syndrome, Cerebral injury, Extracorporeal membrane oxygenation, Femoral cannulation

## Abstract

**Background:**

Differential hypoxemia, or Harlequin syndrome, is a critical concern during veno-arterial extracorporeal membrane oxygenation (VA-ECMO), especially when femoral artery cannulation delivers retrograde flow. This phenomenon may impair cerebral oxygenation and promote brain injury. We investigated whether the arterial cannulation site and ventilation strategy influence acute cerebral injury in a rat cardiopulmonary bypass (CPB) model.

**Methods:**

Forty male Wistar rats were randomized into four groups (*n* = 10 each) according to the arterial cannulation site (carotid [anterograde, A-CPB] or femoral [retrograde, R-CPB]) and ventilation strategy (normal [NV, 100% tidal volume] or low [LV, 50% tidal volume]). All underwent 60 min of CPB at 50–60 mL/kg/min. Systemic hemodynamics, arterial blood gases, and plasma biomarkers of cerebral injury (neuron-specific enolase [NSE] and S100B) were measured. Postmortem brain water content was quantified to assess edema.

**Results:**

Mean arterial pressure and heart rate were comparable across groups. Oxygenation (PaO₂) was preserved, whereas PaCO₂ was higher under low ventilation (A-CPB LV vs. A-CPB NV, *p* = 0.002; R-CPB LV vs. R-CPB NV, *p* = 0.030). Lactate levels increased significantly in the R-CPB LV group compared with the A-CPB NV group (*p* = 0.004). At 60 min, NSE and S100B were markedly elevated in the R-CPB LV group compared with the other groups (*p* < 0.001). Regional brain edema was significantly greater in the cerebellum and left telencephalon of R-CPB LV animals (*p* < 0.001 and *p* = 0.002, respectively), whereas other regions showed no significant changes.

**Conclusions:**

Femoral retrograde perfusion, particularly under reduced ventilation, exacerbates biochemical and structural markers of cerebral injury in a rat CPB model. These findings reproduce key pathophysiological features of Harlequin syndrome and emphasize the importance of proximal cannulation strategies and optimized ventilation to preserve cerebral oxygen delivery during VA-ECMO support.

## Background

Neurological injury is a significant source of morbidity and mortality following veno-arterial extracorporeal membrane oxygenation (VA-ECMO), particularly when arterial perfusion is established via femoral artery cannulation [1, 2]. Such retrograde perfusion strategies can precipitate differential hypoxemia, clinically termed Harlequin syndrome, wherein oxygenated extracorporeal circuit blood returning via the femoral artery inadequately perfuses the upper body and cerebrum because of competitive mixing with desaturated blood [3, 4]. Previous clinical observations and case series have documented substantial neurological morbidity potentially attributable to retrograde arterial perfusion, highlighting the need to elucidate the underlying pathophysiological mechanisms and explore mitigation strategies [1, 5]. Recent experimental investigations have increasingly employed animal models to dissect regional perfusion disparities and associated neuropathological consequences under conditions mimicking ECMO scenarios [6–8]. A rigorously controlled rat cardiopulmonary bypass (CPB) model was used to evaluate whether modification of the arterial cannulation site and anterograde (carotid) versus retrograde (femoral) perfusion under varying simulated ventilation states significantly influenced acute cerebral injury. The primary objective of this study was to provide direct experimental evidence regarding the impact of perfusion strategy on cerebral outcomes in a model pertinent to Harlequin syndrome pathophysiology, thereby informing safer extracorporeal life-support strategies.

## Methods

### Animal care and study groups

This study is reported in accordance with the ARRIVE 2.0 guidelines for reporting animal research. All experimental protocols were approved by the regional animal ethics committee and authorised by the French Ministry of Higher Education and Research, in accordance with EU Directive 2010/63/EU and French Decret no. 2013-118, and complied with the Guide for the Care and Use of Laboratory Animals (8th Edition, National Research Council, National Academies Press, 2011). Male Wistar rats (400–450 g) were housed at a constant temperature of 21 °C under a 14/10-h light/dark cycle with ad libitum access to standard chow and water.

Forty rats were randomly allocated to four experimental groups (*n* = 10 per group): (1) Anterograde CPB with Normal Ventilation (A-CPB NV), (2) Retrograde CPB with Normal Ventilation (R-CPB NV), (3) Anterograde CPB with Low Ventilation (A-CPB LV), and (4) Retrograde CPB with Low Ventilation (R-CPB LV).

### Surgical procedure

**Fig. 1:**
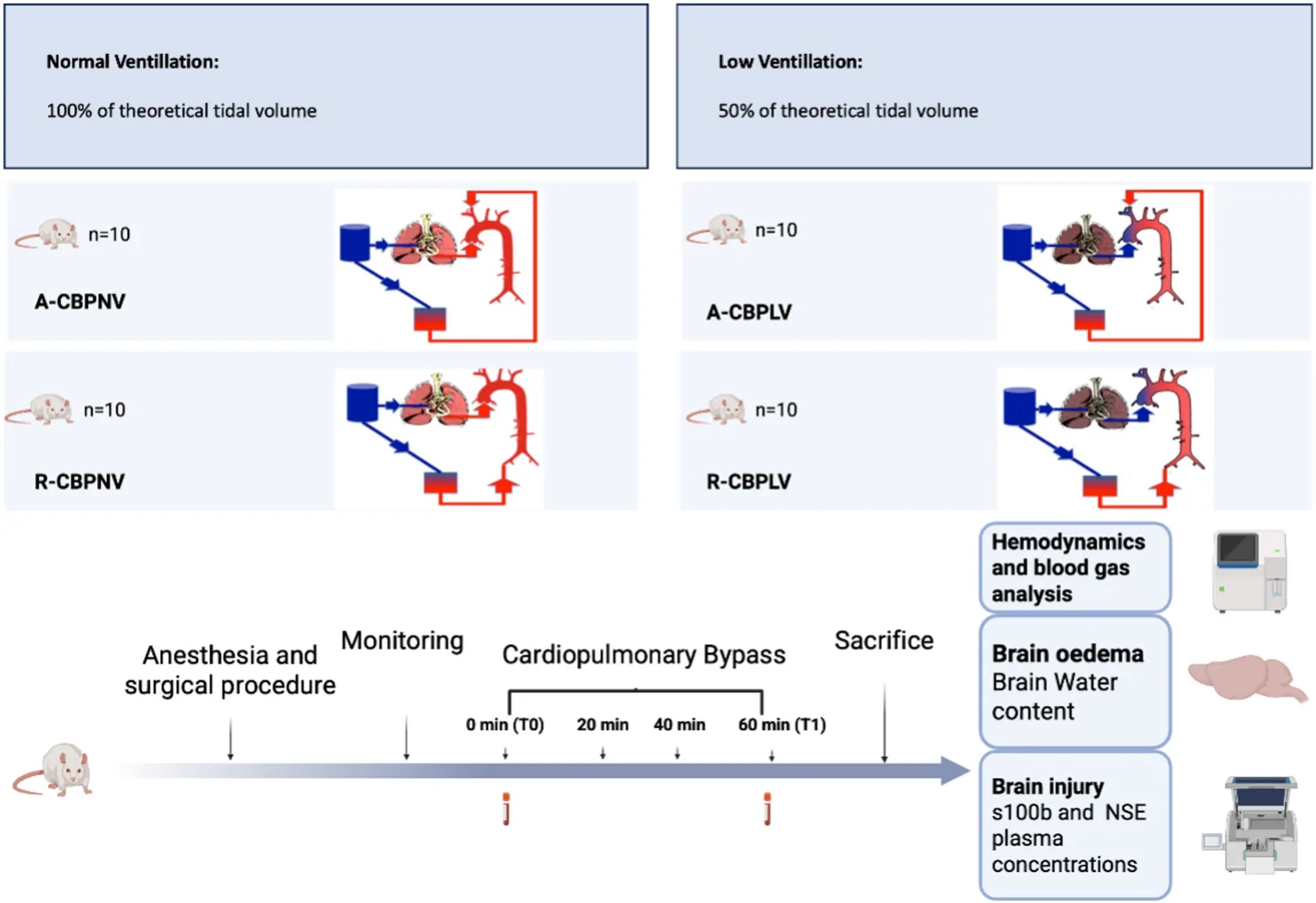
Overview of the study design and experimental timeline. Group allocation under normal ventilation (100% tidal volume) and low ventilation (50% tidal volume), procedural timeline from anesthesia and surgical preparation through CPB (60 min), monitoring points (T0 to T1), sacrifice, and outcome assessments including hemodynamics, blood gas analysis, brain water content, and plasma concentrations of S100B and neuron-specific enolase (NSE)

Anaesthesia was induced via intraperitoneal administration of ketamine (80 mg/kg) and xylazine (10 mg/kg), and maintenance doses of ketamine were administered every 30 min. Following induction, a tracheotomy was performed using a 16-gauge catheter (Introcan Safety, B. Braun, France) to facilitate mechanical ventilation (Rodent ventilator, Model 7025; Ugo Basile, Italy). Ventilation parameters were standardized as follows: respiratory rate, 75 cycles/min; tidal volume, 6 mL/kg. During CPB, ventilation was maintained with 100% oxygen. The normal-ventilation (NV) groups received 100% of the calculated tidal volume, whereas the low-ventilation (LV) groups received 50% of that value to model reduced alveolar ventilation (reduced mechanical convection) at a constant respiratory rate and an inspired oxygen fraction of 100% rather than a protective ventilation strategy. The experimental allocation and assessment workflow are summarized in Fig. 1.

**Fig. 2:**
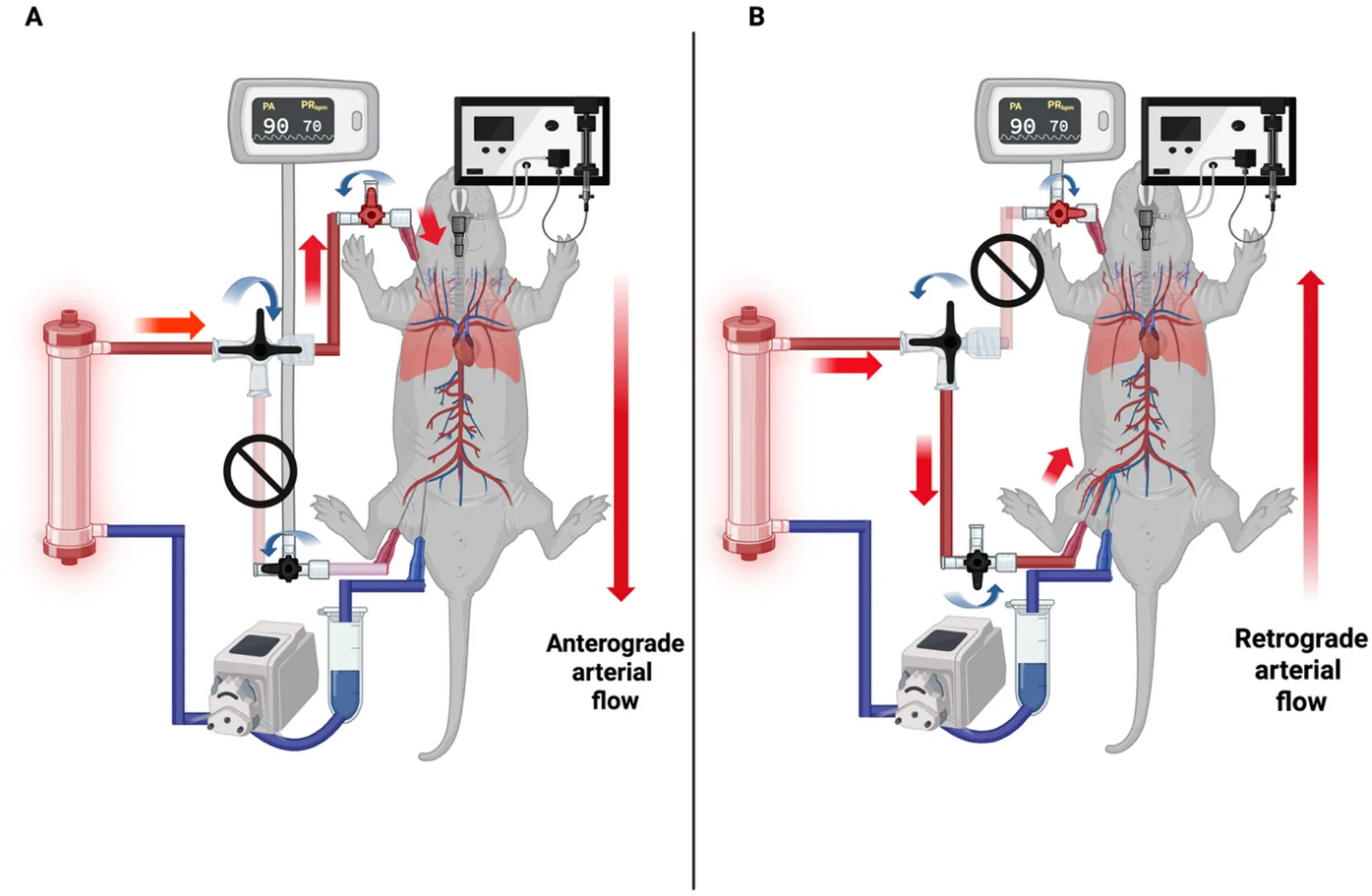
Schematic illustration of arterial cannulation strategies in the rat cardiopulmonary bypass (CPB) model. (**A**) Anterograde arterial flow via carotid artery cannulation, showing oxygenated blood directed cranially with preserved upper-body perfusion. (**B**) Retrograde arterial flow via femoral artery cannulation, depicting potential mixing zones and risk of differential hypoxemia in the upper body. Monitors schematically illustrate representative arterial pressure and oxygenation trends

The right common carotid artery was cannulated with a 22-gauge catheter (Introcan Safety) for continuous arterial pressure and heart rate monitoring using LabChart software (ADInstruments, Colorado, USA). Heparin (500 IU/kg) was intravenously administered before the initiation of CPB. For venous drainage, the right femoral vein was cannulated with a 16-gauge catheter (Introcan Safety). Arterial cannulation varied by group: for anterograde perfusion (A-CPB groups), the right common carotid artery was cannulated with a 22-gauge catheter; for retrograde perfusion (R-CPB groups), the right femoral artery was cannulated with a 22-gauge catheter. The two arterial return configurations are schematically illustrated in Fig. 2. Rectal temperature was monitored (Eroscan Temp 4, Eutech Instruments, Netherlands) and maintained at approximately 37 °C. The rats were positioned at a 30° inclination to optimize venous return. The surgical preparation and cardiopulmonary bypass methodology adhered to protocols previously validated by our research team [7–10].

### Cardiopulmonary bypass circuit

A sterile CPB circuit was employed, comprising a roller pump (Gambro), membrane oxygenator (Micro-1; Kewei Medical Instrument Inc., China), polyvinyl chloride tubing (2.5 mm), and a 5-mL syringe venous reservoir (Terumo, Tokyo, Japan). The circuit was primed with 10 mL of 4% succinylated gelatin solution (Gelofusine; B. Braun, Germany) containing heparin (100 IU/kg). The oxygenator was supplied with 100% oxygen. CPB was initiated and pump flow was progressively increased to a target rate of 50–60 mL/kg/min. Intravascular volume was maintained with supplemental 4% gelatin solution as required to keep mean arterial pressure above 55 mmHg. No blood transfusions were administered. A system of three-way stopcocks allowed switching between the carotid and femoral arterial lines according to group allocation while maintaining circuit consistency.

### Blood gas analysis

Arterial blood samples (0.2 mL) were collected at baseline (T0, pre-CPB) and at 20, 40, and 60 min (T1) during CPB. Samples were immediately analyzed using an ABL 700 series blood gas analyzer (Radiometer, Copenhagen, Denmark) for the partial pressures of oxygen (PaO₂) and carbon dioxide (PaCO₂), pH, bicarbonate concentration (HCO₃⁻), lactate levels, and hematocrit (Hct).

### Brain water content assessment

At T1, brains were rapidly excised and dissected into five distinct regions: cerebellum, right diencephalon, right telencephalon, left diencephalon, and left telencephalon. Each region was immediately weighed to obtain the wet weight (Sartorius LA 230 P, Goettingen, Germany). Samples were desiccated in a vacuum oven (Memmert, Schwabach, Germany) at 100 °C until a constant dry weight was achieved. Brain water content (BWC) was calculated using the standard wet-to-dry weight method, as previously described [11, 12], according to the following formula: %H₂O = [(wet weight − dry weight) / wet weight] × 100.

### Quantification of plasma NSE and S100B concentrations

Blood samples (1 mL) were collected in EDTA tubes at T0 and T1. Plasma was separated by centrifugation (3000 rpm, 5 min, 4 °C) and stored at − 80 °C until analysis. Plasma concentrations of neuron-specific enolase (NSE) and S100B protein were quantified using commercially available ELISA kits according to the manufacturers’ protocols (NSE: USCN Life Science Inc., USA; S100B: MyBioSource, USA).

### Statistical analysis

Data are presented as mean ± standard deviation (SD). Statistical comparisons for serially measured parameters (hemodynamics and blood gases) were performed using repeated-measures ANOVA followed by Tukey’s post hoc test where appropriate. Endpoint measurements (biomarkers and brain water content) were compared using one-way or two-way ANOVA followed by Tukey’s post hoc test. Statistical significance was set at *p* < 0.05. All analyses were conducted using GraphPad Prism software (version 10; GraphPad Software, USA).

## Results

### Hemodynamic parameters

**Fig. 3:**
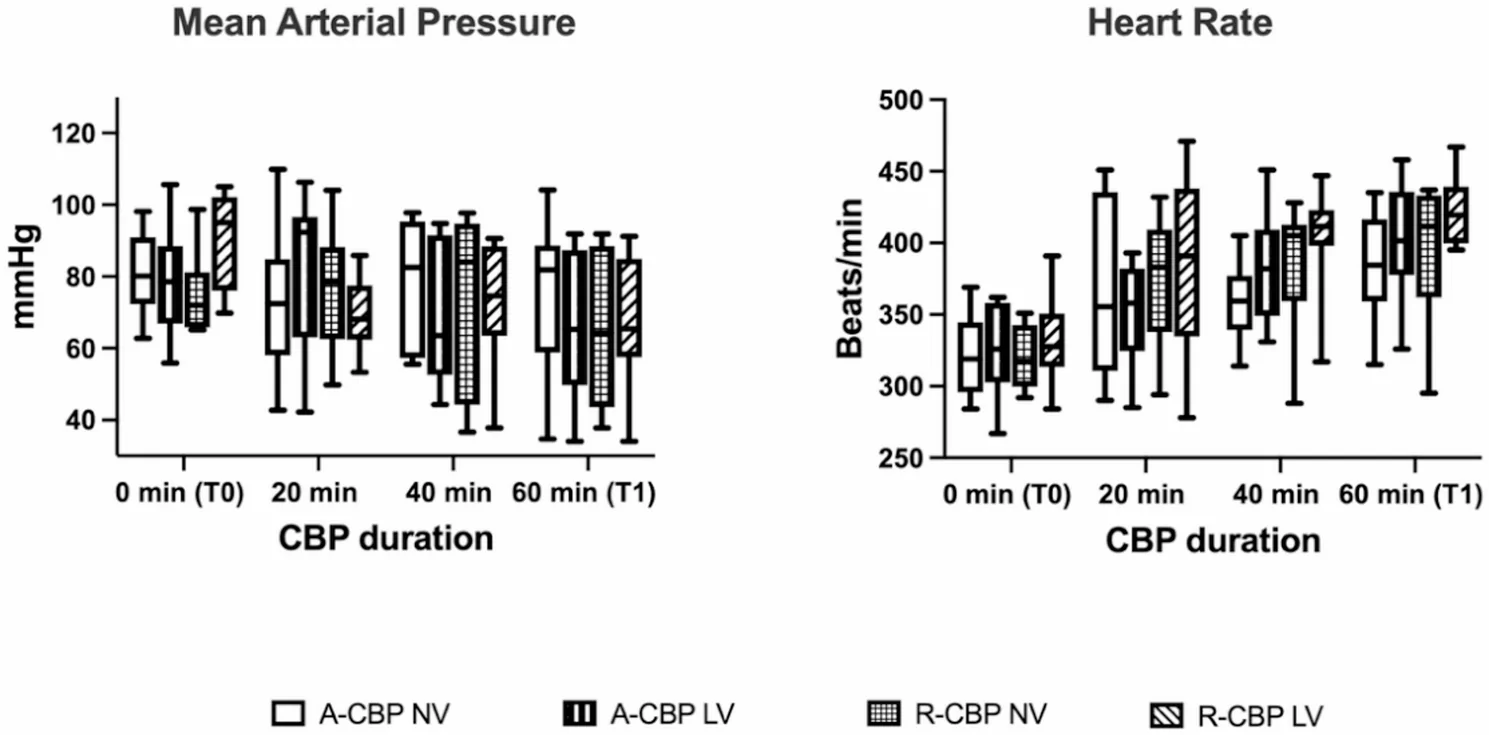
Hemodynamic parameters during cardiopulmonary bypass. Line graphs depict mean arterial pressure (MAP, mmHg) and heart rate (HR, bpm) measured at baseline (T0), 20, 40, and 60 min (T1) during CPB in A-CPB NV, A-CPB LV, R-CPB NV, and R-CPB LV groups (*n* = 10 per group). MAP remained stable across groups, and HR increased gradually over time without significant intergroup differences

Mean arterial pressure (MAP) and heart rate (HR) were continuously monitored throughout the 60-min CPB period (Fig. 3). MAP showed no statistically significant variation among the four groups at T0, 20 min, 40 min, or T1 (60 min). Similarly, although HR tended to increase over time in all groups, no significant intergroup differences were observed at any time point. These data indicate comparable systemic hemodynamic stability across the experimental conditions.

### Arterial blood gas analysis

**Fig. 4:**
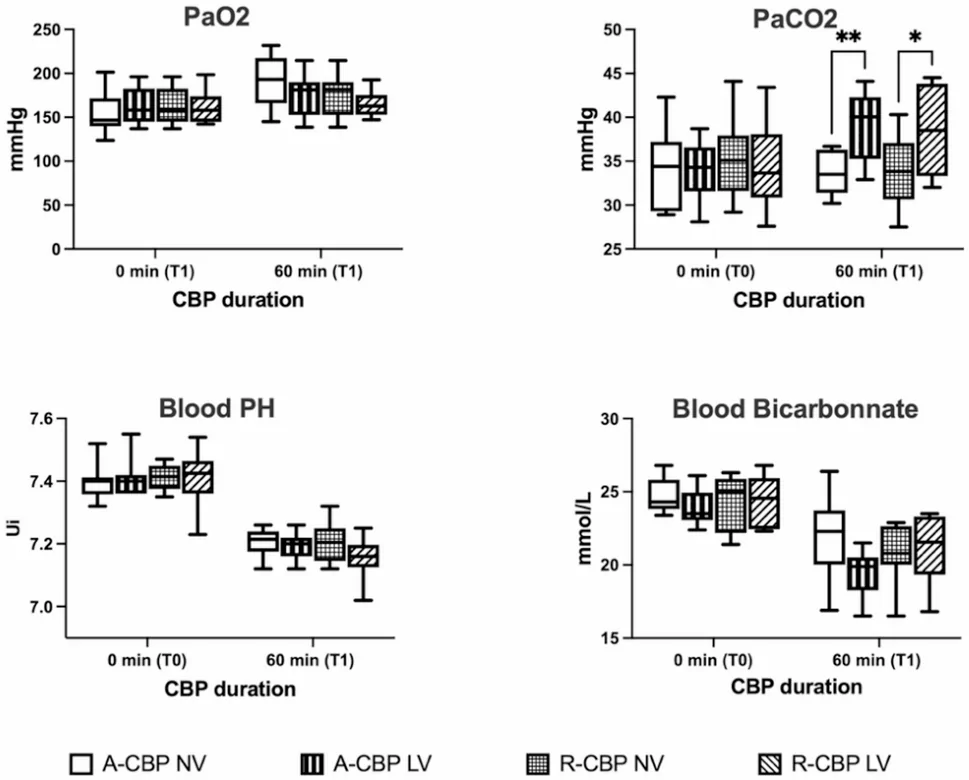
Arterial blood gas parameters during cardiopulmonary bypass (CPB) at T0, 20, 40 and 60 min (T1). Top-left: PaO₂ (mmHg). Top-right: PaCO₂ (mmHg). Bottom-left: blood pH. Bottom-right: bicarbonate (mmol/L). Groups: A-CPB NV (open bars), A-CPB LV (vertical hatching), R-CPB NV (grid), and R-CPB LV (diagonal hatching). Data are presented as mean ± SD (*n* = 10/group). *p < 0.05, **p < 0.01 for PaCO₂ increases under low-ventilation strategies

**Fig. 5:**
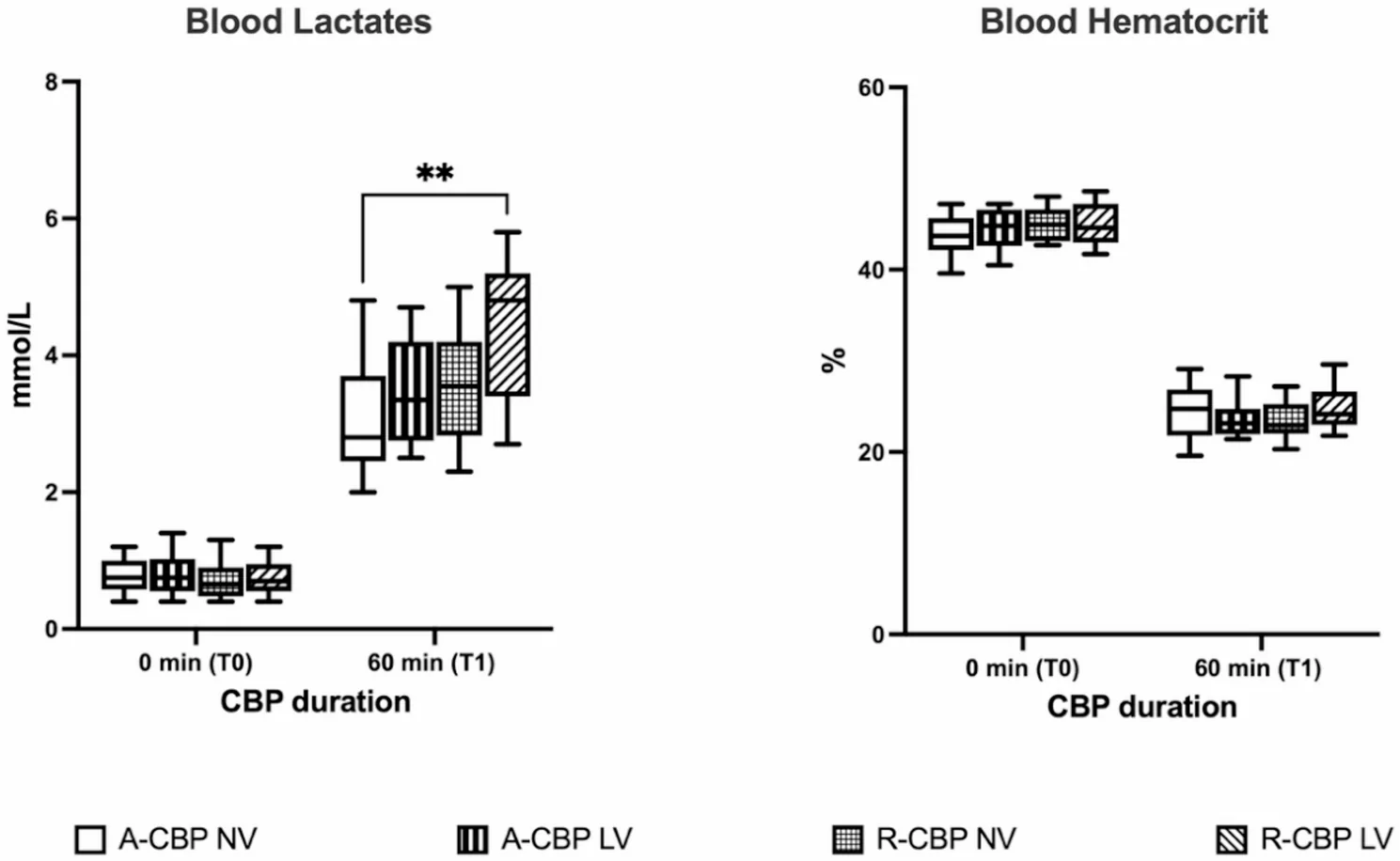
Metabolic surrogates during cardiopulmonary bypass (CPB). Left: circulating lactate concentrations (mmol/L) at T0 and T1. Right: blood hematocrit (%) at T0 and T1. Lactate increased significantly in the R-CPB LV group compared with the A-CPB NV group at T1 (**p < 0.01). Box-and-whisker plots show median, interquartile range, and whiskers for A-CPB NV (open boxes), A-CPB LV (vertical hatching), R-CPB NV (grid), and R-CPB LV (diagonal hatching)

Arterial blood gas parameters were assessed serially (Fig. 4). PaO₂ remained adequately maintained and did not differ significantly among groups. Conversely, PaCO₂ diverged according to ventilation strategy. At T1, PaCO₂ was significantly higher in the low-ventilation groups than in their normal-ventilation counterparts (A-CPB LV vs. A-CPB NV, *p* = 0.002; R-CPB LV vs. R-CPB NV, *p* = 0.030). Arterial pH showed a transient difference at 40 min between the A-CPB NV and R-CPB LV groups (*p* = 0.049), which resolved by T1 and did not survive correction for multiple comparisons. Bicarbonate concentrations gradually declined over time in all groups, consistent with hemodilution and buffering, without significant intergroup differences. Blood lactate at T1 was significantly higher in the R-CPB LV group than in the A-CPB NV group (*p* = 0.004). Hematocrit decreased comparably in all groups from T0 to T1, with no significant intergroup differences at T1 (Fig. 5).

### Plasma concentrations of S100B and NSE

**Fig. 6:**
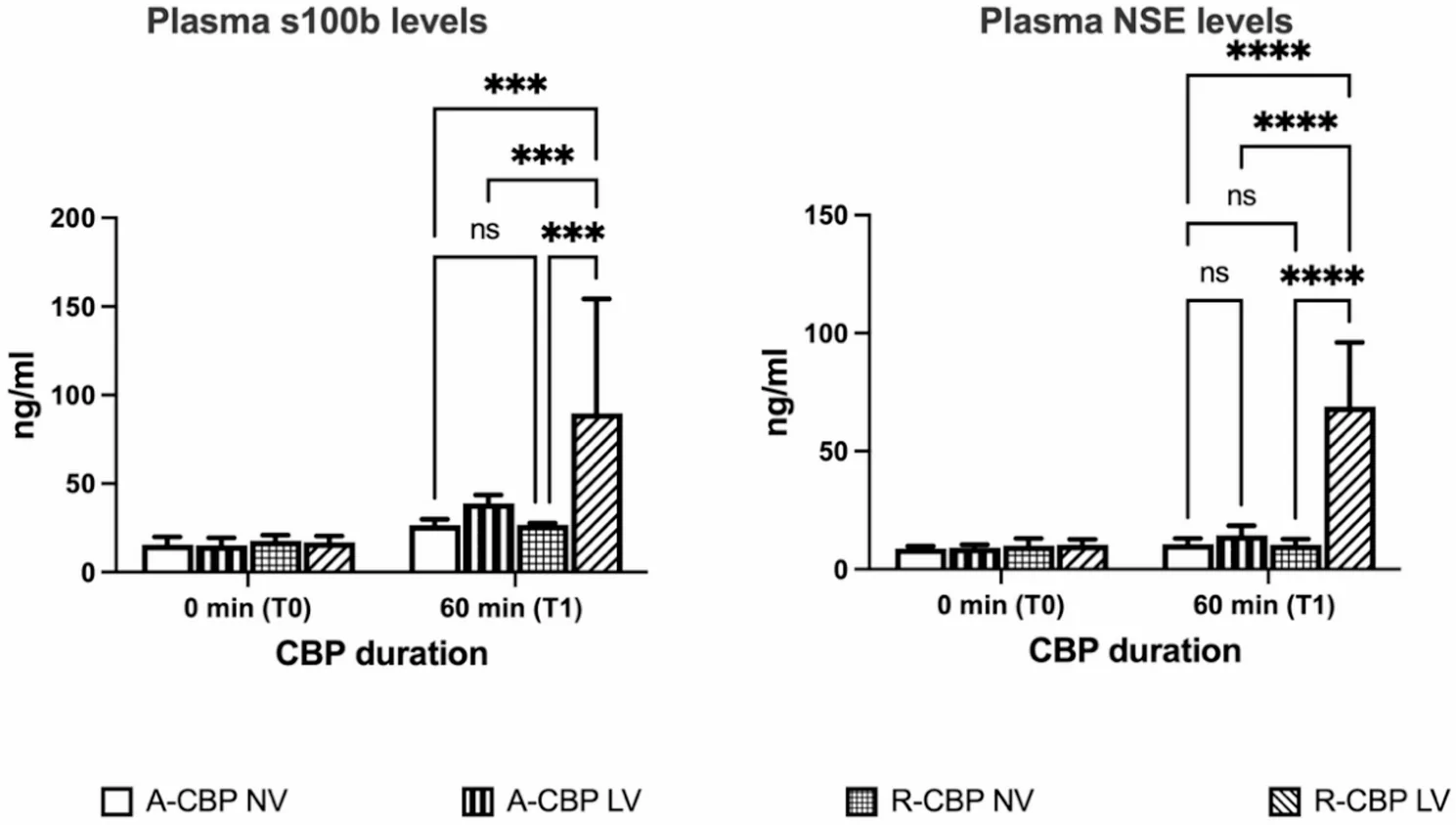
Plasma biomarkers of cerebral injury during cardiopulmonary bypass (CPB). Left: S100B concentrations (ng/mL) at T0 and T1. Right: neuron-specific enolase (NSE) concentrations (ng/mL) at T0 and T1. Both biomarkers were significantly increased in the R-CPB LV group at T1 compared with the other groups. Box-and-whisker plots show median, interquartile range, and whiskers for A-CPB NV, A-CPB LV, R-CPB NV, and R-CPB LV

Plasma concentrations of the brain injury biomarkers S100B and NSE were quantified at baseline (T0) and 60 min after CPB (T1) (Fig. 6). At T0, no significant intergroup differences were observed in S100B or NSE levels. At T1, plasma S100B concentrations were significantly elevated in the R-CPB LV group compared with the A-CPB NV group (*p* < 0.001). Similarly, NSE levels were significantly higher in the R-CPB LV group than in the other groups (*p* < 0.001).

### Regional brain water content

**Fig. 7:**
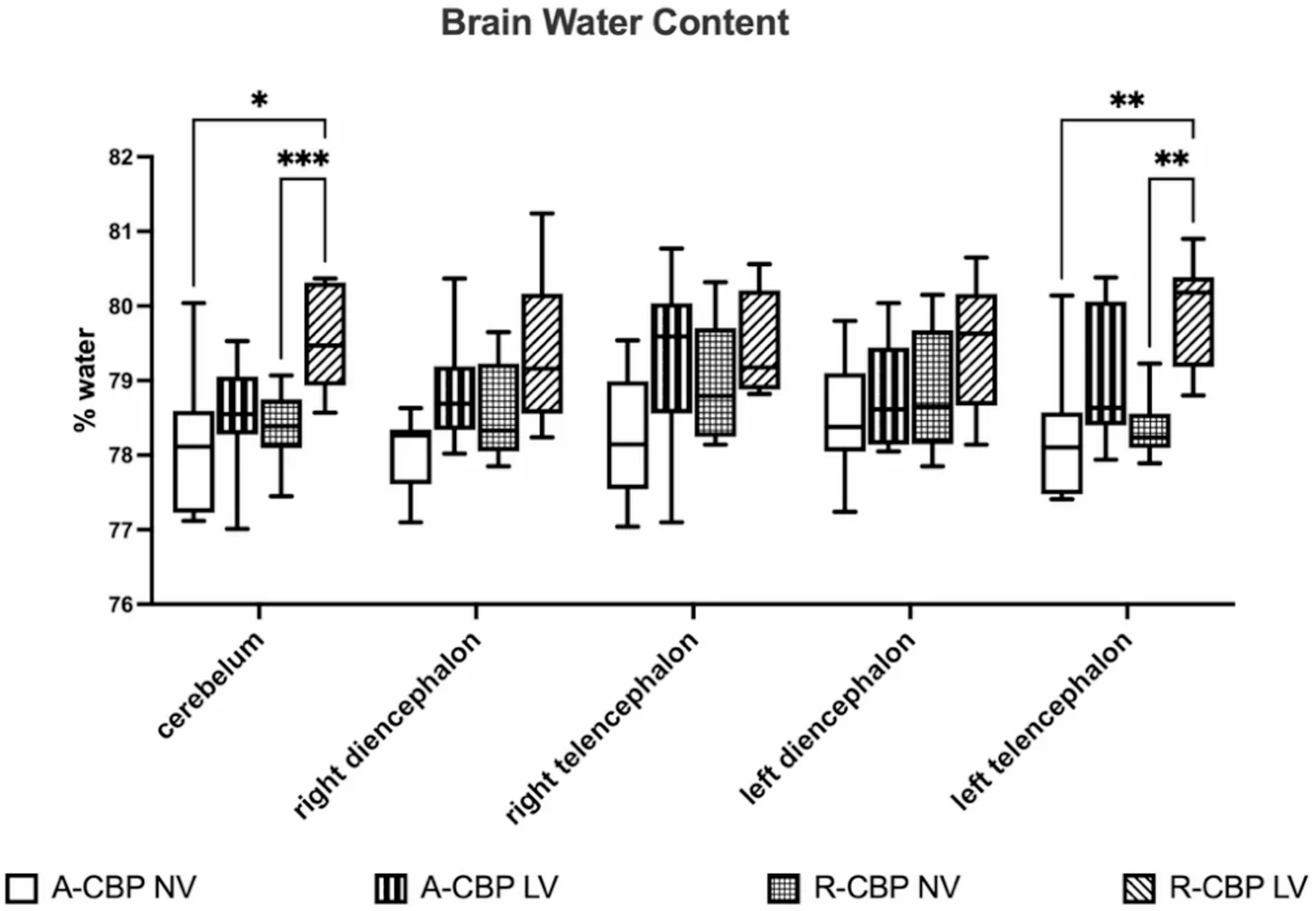
Regional brain water content after cardiopulmonary bypass (CPB). Brain water content was significantly increased in the cerebellum and left telencephalon in the R-CPB LV group. Box-and-whisker plots show median, interquartile range, and whiskers for each group

Regional brain water content was assessed after CPB (Fig. 7). In the cerebellum, BWC was significantly higher in the R-CPB LV group than in the A-CPB NV (*p* = 0.040) and R-CPB NV (*p* < 0.001) groups. In the left telencephalon, BWC was significantly elevated in the R-CPB LV group compared with the A-CPB NV and R-CPB NV groups (*p* = 0.002). No significant differences were observed in the right diencephalon, right telencephalon, or left diencephalon.

## Discussion

### Principal findings and interpretation

The present study addressed whether the arterial cannulation site (femoral vs. carotid), in interaction with the ventilation strategy, modulates the extent of acute cerebral injury during CPB in a rat model relevant to Harlequin syndrome. Our findings indicate that retrograde perfusion, particularly under low ventilation, exacerbates cerebral injury. This conclusion is supported by convergent evidence: (i) markedly elevated plasma concentrations of NSE and S100B in the R-CPB LV group; (ii) increased regional brain water content, indicating edema, predominantly in the cerebellum and left telencephalon; and (iii) higher lactate levels in the R-CPB LV group, suggesting metabolic stress consistent with impaired regional perfusion. These results support the concept that retrograde perfusion, when combined with reduced alveolar ventilation, potentiates acute cerebral injury compared with anterograde perfusion strategies.

These experimental data reinforce clinical concerns regarding neurological complications associated with peripheral VA-ECMO using femoral artery cannulation [1, 2, 13, 14]. Differential hypoxemia arises from competitive aortic flow dynamics between retrograde oxygenated ECMO return and antegrade native ventricular ejection, particularly in the setting of concomitant respiratory failure [3, 4, 15]. Such dynamics can lead to preferential exposure of the cerebral and coronary circulations to inadequately oxygenated blood [16]. Our study provides in vivo experimental evidence of the detrimental neurological consequences associated with this retrograde flow pattern under reduced alveolar ventilation.

Rodent models are increasingly used to investigate CPB and ECMO pathophysiology because they provide strong experimental control and mechanistic resolution [6, 7, 17]. Previous rat CPB models have explored inflammatory responses, hemodilution, and neurological dysfunction [18]. Lebreton et al. developed a minimally invasive femoro-femoral CPB model [7], and Mackensen and colleagues demonstrated neurological and neurocognitive dysfunction after CPB in rats [17]. More recently, multimodal characterization of brain injury evolution after cardiac arrest has been described in rats [19]. Our study builds on these precedents by introducing a factorial comparison of anterograde versus retrograde perfusion under different ventilation conditions, thereby isolating the interaction that is most relevant to differential hypoxemia.

In the aorta, the position of the mixing zone between oxygenated extracorporeal return and antegrade native left-ventricular ejection is a key determinant of supra-aortic and coronary oxygenation. During femoral retrograde perfusion, the extracorporeal flow front meets native ejection in the descending or distal aorta, generating a counter-directed interface whose proximal extent depends on the balance between extracorporeal flow, residual cardiac output, and the oxygen content of native ejected blood; this retrograde column also opposes left-ventricular ejection, increasing aortic-valve loading and favoring ventricular distension. By contrast, carotid return in the present model does not act through antegrade aortic flow but through direct supra-aortic delivery: oxygenated circuit blood enters a cervical trunk and perfuses the cerebral circulation cranially, downstream of and in parallel with the aortic watershed, thereby decoupling cerebral oxygenation from both the aortic mixing zone and native pulmonary gas exchange. This dual effect — preserved cerebral oxygen delivery and reduced ascending-aortic afterload — provides a coherent mechanism for the lower biomarker release and regional edema observed in the A-CPB groups, analogous to the rationale for axillary and innominate cannulation and antegrade cerebral perfusion in arch surgery [20]; because the caliber of these vessels precludes their cannulation in the rat, the carotid route is best understood as an experimental surrogate for proximal anterograde return rather than as a clinical strategy reproducible in humans. Because native cardiac output, the exact mixing-zone location, and aortic-valve loading were not directly measured, these mechanisms are presented as physiologically plausible and consistent with prior computational and experimental work [20–23] rather than as direct proof of a clinical mechanism.

The significant elevation of plasma NSE and S100B in the R-CPB LV group is consistent with their established roles as biomarkers of neuronal and astroglial injury in acute brain injury and extracorporeal support settings [24–26]. The magnitude of these increases suggests that substantial acute cellular injury was triggered by the combination of retrograde perfusion and hypoventilation. In clinical ECMO cohorts, elevated S100B has been associated with adverse neurological events, although interpretation remains susceptible to confounding [25]. In the present controlled model, biomarker release was directly associated with a defined perfusion–ventilation mismatch, strengthening the mechanistic interpretation.

Increased regional brain water content in the cerebellum and left telencephalon of the R-CPB LV group, assessed using the dry/wet weight method [12], indicates region-specific vulnerability under these conditions. Whereas global hypoxic-ischemic injury often emphasizes hippocampal susceptibility [27], the present pattern suggests that simulated differential hypoxemia may produce a distinct spatial distribution of injury, potentially related to regional vascular supply, competitive flow fronts, and differing metabolic demands.

Critically, the observed differences in cerebral injury biomarkers and lactate occurred despite the absence of major differences in global systemic hemodynamics among groups. These findings suggest that systemic hemodynamic stability does not necessarily reflect homogeneous regional perfusion under peripheral VA-ECMO-like flow conditions. Our results are therefore more consistent with altered perfusion distribution during retrograde flow in the setting of reduced alveolar ventilation than with direct proof of cerebral hypoxemia. This interpretation is consistent with prior experimental and computational work showing that arterial cannula position and aortic flow-mixing patterns are major determinants of supra-aortic perfusion during peripheral VA-ECMO [20–23].

### Strengths and limitations

This study has several strengths. It used a randomized design comparing four clinically relevant experimental conditions in a validated rat CPB model, allowing rigorous control of perfusion and ventilation variables. In addition, cerebral injury was assessed using a multimodal approach combining systemic hemodynamics, arterial blood gases, established plasma biomarkers, and quantitative brain water content. This integrated design enhances the translational relevance of the model by reproducing key pathophysiological features associated with Harlequin syndrome.

Several limitations should nevertheless be acknowledged. First, in this model, the low-ventilation strategy induced hypercapnia more prominently than frank systemic hypoxemia because animals remained ventilated with 100% oxygen. Accordingly, the model reproduced reduced alveolar ventilation with hypercapnia rather than frank systemic arterial hypoxemia and should be considered a simplified experimental surrogate rather than a full reproduction of the clinical spectrum of Harlequin syndrome. Because PaO₂ remained preserved across groups, the present design did not reproduce systemic arterial hypoxemia, and no conclusions regarding the effects of frank hypoxia can be drawn from this model. Hypercapnia itself may still have contributed to cerebral injury through respiratory acidosis, cerebral vasodilation, altered autoregulation, blood–brain barrier disruption, and edema formation, particularly when combined with unfavorable regional perfusion. Second, native cardiac output was not directly quantified, and echocardiography was not performed; therefore, the interaction between native ventricular ejection and extracorporeal flow could not be precisely characterized despite preserved systemic hemodynamic stability across groups. In this context, stable mean arterial pressure does not exclude heterogeneous regional perfusion, and the selective lactate increase observed in the R-CPB LV group is consistent with regional metabolic stress under retrograde flow conditions. Third, right common carotid cannulation in the anterograde groups may theoretically have influenced ipsilateral cerebral perfusion. However, no clear hemispheric asymmetry was observed overall, although the greater edema detected in the left telencephalon and cerebellum in the retrograde low-ventilation group warrants cautious interpretation and further mechanistic investigation. Fourth, the 60-min CPB duration was designed to capture acute pathophysiological changes and does not reproduce the prolonged exposure encountered during clinical VA-ECMO support; accordingly, the present findings primarily reflect early injury mechanisms rather than delayed neurological sequelae [19]. In addition, the assessment was limited to acute biochemical and structural markers of brain injury, without long-term neurological, behavioral, or neuropathological evaluation. Finally, inherent interspecies differences between rodents and humans, together with scaling differences in cannula size and flow conditions, limit direct extrapolation of absolute flow behavior and regional perfusion patterns to clinical practice [28, 29]. This limitation is particularly relevant because arterial cannula position and aortic flow-mixing dynamics are major determinants of supra-aortic perfusion during peripheral VA-ECMO [22, 23].

A point of translational interpretation warrants explicit emphasis. Carotid arterial cannulation is not employed as an arterial-return strategy for mechanical circulatory support in adult patients; clinically, proximal anterograde return is achieved through axillary/subclavian, innominate, or central aortic cannulation. Because the caliber of these vessels precludes their cannulation in the rat, the common carotid was used here as a methodological surrogate for proximal anterograde arterial return, and the resulting configuration approximates axillary or central aortic cannulation rather than representing a clinical carotid strategy. Consequently, this model is not, as such, reproducible in humans: it isolates the effect of the site and direction of arterial return on cerebral oxygenation, while the literal carotid route — particularly if occlusive and without distal reperfusion — would be expected to cause low cerebral flow, regional hypoxia, and cytotoxic edema in the ipsilateral hemisphere. Our findings should therefore be interpreted as supporting proximal, anterograde cannulation strategies (axillary or central) rather than carotid cannulation per se.

The applicability of the cannulation geometry to humans should also be considered in terms of the cannula-to-artery diameter ratio and the resulting reduction in cross-sectional area, functionally analogous to an arterial stenosis. A 15–17 Fr adult cannula (approximately 5–6 mm) within a common carotid of approximately 6–6.5 mm occupies most of the lumen and would be virtually occlusive for residual native flow, whereas in the rat the cranially-directed 22-G cannula substitutes circuit flow for native carotid flow, making collateral sufficiency rather than residual lumen the operative variable. The contralateral carotid axis and the vertebro-basilar contribution to the circle of Willis are additional determinants that differ between model and clinic. Experimental studies of unilateral carotid ligation in the rat indicate that collateral adaptation preserves cerebral blood flow and energy metabolism without overt structural injury [30, 31], consistent with the absence of major hemispheric asymmetry in our study.

The absence of direct cardiac-performance data constrains mechanistic interpretation. Arterial pressure and heart rate were continuously recorded and remained comparable across groups, but these signals cannot substitute for direct assessment of contractility, native cardiac output, or ventricular loading; the recordings were acquired for pressure and heart-rate monitoring and did not permit reliable derivation of an arterial dP/dt index, and echocardiography and conductance-catheter measurements were not obtained. Consequently, the relative contributions of retrograde arterial return and of venous-drainage–related preload reduction to cerebral injury — both recognized mediators of North–South physiology — cannot be fully disentangled, and the fraction of retrograde flow reaching the cerebral circulation could not be quantified. Future experiments should incorporate serial echocardiography, left-ventricular pressure–volume analysis, high-fidelity arterial-waveform and dP/dt acquisition, cerebral near-infrared spectroscopy, and cerebral blood-flow monitoring.

### Implications for practice and future research

These findings support heightened vigilance when femoral arterial cannulation is used in patients at risk of differential hypoxemia, particularly in the setting of reduced alveolar ventilation. Alternative arterial cannulation sites such as the axillary/subclavian artery or central aorta may be considered to reduce the risk of upper-body hypoxemia [20]. Our findings also support the use of multimodal monitoring strategies, including cerebral near-infrared spectroscopy, coupled with careful optimization of native pulmonary function and cardiac output [32, 33].

Future studies should evaluate longer durations of extracorporeal support, multimodal neuromonitoring, and the potential impact of VAV-ECMO conversion in this setting [15]. Larger-animal models may also help extend perfusion duration and facilitate integration of clinical-grade monitoring and neuroimaging approaches [6].

## Conclusion

Using a controlled rat cardiopulmonary bypass model relevant to Harlequin syndrome, this study demonstrates that retrograde arterial perfusion via femoral cannulation, particularly when combined with reduced ventilation, significantly exacerbates acute cerebral injury compared with anterograde perfusion strategies. These findings highlight the importance of arterial cannulation site and flow direction in determining neurological risk during extracorporeal support and support vigilant monitoring and optimized cannulation strategies to enhance cerebral protection during VA-ECMO.

### Take-home message

Retrograde femoral perfusion combined with low ventilation in a rat CPB model increases cerebral injury biomarkers and regional brain edema, reproducing key pathophysiological features of Harlequin syndrome. Proximal cannulation and optimized ventilation may help protect the brain during VA-ECMO.

## Data Availability

The datasets generated and analyzed in the current study are available from the corresponding author upon reasonable request. All relevant raw data, including hemodynamic recordings, blood gas measurements, biomarker concentrations, and brain water content values, have been archived and can be shared in an anonymized format for non-commercial academic purposes.

## Abbreviations

A-CPB LV: Anterograde cardiopulmonary bypass with low ventilation
A-CPB NV: Anterograde cardiopulmonary bypass with normal ventilation
ANOVA: Analysis of variance
BWC: Brain water content
CPB: Cardiopulmonary bypass
ECMO: Extracorporeal membrane oxygenation
EDTA: Ethylenediaminetetraacetic acid
ELISA: Enzyme-linked immunosorbent assay
Hct: Hematocrit
HCO₃⁻: Bicarbonate concentration
HR: Heart rate
LV: Low ventilation
MAP: Mean arterial pressure
NIRS: Near-infrared spectroscopy
NSE: Neuron-specific enolase
NV: Normal ventilation
PaCO₂: Partial pressure of arterial carbon dioxide
PaO₂: Partial pressure of arterial oxygen
R-CPB LV: Retrograde cardiopulmonary bypass with low ventilation
R-CPB NV: Retrograde cardiopulmonary bypass with normal ventilation
SD: Standard deviation
VA-ECMO: Veno-arterial extracorporeal membrane oxygenation
VAV-ECMO: Veno-arterial-venous extracorporeal membrane oxygenation
